# Avian Influenza Prevalence in Pigs, Egypt

**DOI:** 10.3201/eid1604.091316

**Published:** 2010-04

**Authors:** Amr El-Sayed, Walid Awad, Adel Fayed, Hans-Peter Hamann, Michael Zschöck

**Affiliations:** Cairo University, Giza, Egypt (A. El-Sayed, W. Awad, A. Fayed); Hessian State Laboratory, Giessen, Germany (H.P. Hamann, M. Zschöck)

**Keywords:** Avian influenza, Egypt, H5N1, hemagglutinating inhibition test, real-time PCR, swine, influenza, viruses, letter

**To the Editor:** Since 1996, avian influenza virus (H5N1) has spread to >65 countries (*1*). The disease represents a serious threat for the poultry industry and public health. Egypt has the highest human infection and fatality rates outside Asia (*2*). Some isolates of influenza virus (H5N1) in Egypt are resistant to oseltamivir (*3*), and in others, virulent mutations have developed, leading to case-fatality rates of 100% (*4*).

Pigs have the largest epidemiologic role in the evolution of new influenza viruses (*5*). Recombination between the newly emerged influenza virus subtypes H1N1 and H5N1 in pigs would have catastrophic results. We therefore investigated the seroprevalence of influenza virus (H5N1) in pigs in Egypt.

In May 2008, we collected 1 serum sample and 1 nasal swab from each of 240 pigs (11 herds) in Cairo slums. May was selected because it directly follows the season of bird migration and the seasonal storms usually accompanied by airborne diseases. Cairo slums were selected because 1) pigs there feed on organic remains, including dead birds, and thus have a higher chance of becoming infected; 2) Cairo is at the base of the Nile Delta, where most subtype H5N1 foci occurred; and 3) Cairo is near Fayum, the main stopover site for migrating birds.

To detect anti–avian influenza antibodies in the serum, we used hemagglutination inhibition (HI) assays with 2 inactivated antigens: subtype H5N2 from the Veterinary Laboratories Agency, UK; and a local subtype H5N1 prepared according to the protocol used in the central national laboratories. To detect viral RNA in the nasal swabs, we used real-time PCR, as was recommended for detection of influenza (H5N1) infection during outbreaks in Southeast Asia (*6*).

Although all nasal samples reacted negatively to influenza A/H5 by real-time PCR, only 4 serum samples showed positive results by HI when using subtype H5N2 antigen; titers were 32 for 3 samples and 64 for 1. Seven additional positive serum samples were detected when antigen prepared from local subtype H5N1 virus was used; titers ranged from 16 (6 samples) to 512 (1 sample). Also during this 2-week sampling period, titers of 32 for 3 samples and 128 for 1 were obtained. Seroprevalence rate of avian influenza for the 240 pigs was 1.67% and 4.6% when the nonlocal or local viral antigens, respectively, were used. Of the 11 positive pigs, 8 were from 1 herd and 3 were from 3 other herds.

Failure to detect viral RNA in the upper respiratory tract indicates the absence of acute infections in the investigated pigs. Inability of the virus to persist in the pigs was reported (*7*). Contrary to the HI results, results of routine examination of the 240 pigs found no abnormalities. Absence of clinical signs in infected pigs was reported (*8*) and was attributed to their low susceptibility to influenza (H5N1) (*7*). The results indicate that infection rate for pigs in Egypt is clearly higher than that for pigs in China and Vietnam (*8*,*9*). This increase may be attributed to different spatial and temporal factors leading to increased infection risk among sampled pigs**,** higher antigenicity of native isolates, or most probably to the disease situation in Egypt. The detection of 8 positive reactors from 1 herd indicates a subtype H5N1 focus there as was reported in Indonesia (*8*). The difference in the number of reactors when using different antigens indicates the difference in antigenicity. These data are supported by field observations regarding low protection level (≈35% in some reports) of imported vaccines (A. El-Sayed, unpub. data). The relatively low seroprevalence of avian influenza in pigs may be misleading because of the poor immunogenicity of some avian influenza lines and lack of sensitivity for detecting low titers of induced antibodies (*10*). It may be also explained by the use of a virus antigen other than that existing in the population, as was done in the present study.

Human risk for influenza (H5N1) infection in Egypt seems to be associated mainly with infected birds. It has not yet been associated with infected pigs.
